# ACE I/D genotype, adiposity, and blood pressure in children

**DOI:** 10.1186/1475-2840-8-14

**Published:** 2009-03-16

**Authors:** Joey C Eisenmann, Mark A Sarzynski, Kim Glenn, Max Rothschild, Kate A Heelan

**Affiliations:** 1 Michigan State University, Department of Kinesiology, East Lansing, Michigan, USA; 2 Iowa State University, Department of Animal Science, Ames, Iowa, USA; 3 University of Nebraska-Kearney, Department of Health and Human Performance, Kearney, Nebraska, USA

## Abstract

**Background:**

Angiotensin converting enzyme (ACE) is a possible candidate gene that may influence both body fatness and blood pressure. Although several genetic studies have been conducted in adults, relatively few studies have examined the contribution of potential candidate genes, and specifically ACE I/D, on adiposity and BP phenotypes in childhood. Such studies may prove insightful for the development of the obesity-hypertension phenotype early in life. The purpose of this study was to examine differences in body fatness and resting blood pressure (BP) by ACE I/D genotype, and determine if the association between adiposity and BP varies by ACE I/D genotype in children.

**Methods:**

152 children (75 girls, 77 boys) were assessed for body composition (% body fat) using dual energy x-ray absorbtiometry and resting BP according to American Heart Association recommendations. Buccal cell samples were genotyped using newly developed PCR-RFLP tests for two SNPs (rs4341 and rs4343) in complete linkage disequilibrium with the ACE I/D polymorphism. Partial correlations were computed to assess the ociations between % body fat and BP in the total sample and by genotype. ANCOVA was used to examine differences in resting BP by ACE I/D genotype and fatness groups.

**Results:**

Approximately 39% of youth were overfat based on % body fat (>30% fat in girls, 25% fat in boys). Body mass, body mass index, and fat-free mass were significantly higher in the ACE D-carriers compared to the II group (p < 0.05). BP was not significantly different by ACE I/D genotypes. In the total sample, correlations between adiposity and BP ranged from 0.30 to 0.46, and were not significantly different between genotypes. When grouped by genotype and body fat category, the overfat D-carrier subjects had significantly higher SBP and MAP compared to the normal fat D-carrier and normal fat II groups (p < 0.05).

**Conclusion:**

ACE D-carriers are heavier than ACE II children; however, BP did not differ by ACE I/D genotype but was adversely influenced in the overfat D-carriers. Further studies are warranted to investigate the genetics of fatness and BP phenotypes in children.

## Introduction

Currently, there is considerable interest in the genetics of complex human phenotypes such as obesity and hypertension. The estimated heritability of adiposity measures range from 25–40% [1] and several candidate genes have been identified for obesity phenotypes [2]. The heritability of blood pressure (BP) phenotypes is estimated to be about 30% [1]. A recent genomic scan showed that a number of chromosomal regions have been identified for the phenotypic expression of BP [3]. Although some of these chromosomal regions are specific to BP, other regions also involve genes related to obesity which suggests pleiotropy. An earlier study of children also indicated that a major gene may affect both body mass index and BP [4].

Angiotensin converting enzyme (ACE) is a possible candidate gene that may have pleiotropic effects. ACE is an important regulatory enzyme of the renin-angiotensin-aldosterone (RAA) system, which is a complex system that plays a critical role in maintaining blood pressure homeostasis. ACE converts inactive angiotensin I into active angiotensin II (vasoconstrictor) and inactivates bradykinin and kallidin (vasodilators). Thus, activation of the RAA system results in a vasopressor response mainly through the actions of ACE. The ACE gene is located at 17q23 and contains a polymorphism distinguished by either an insertion (I) or deletion (D) of a 287 base pair segment in intron 16. The ACE DD genotype has been associated with higher levels of ACE [5], BP [3,6-8], fatness [2], and increased cardiovascular risk [5]. Although several genetic studies have been conducted in adults, relatively few studies have examined the contribution of potential candidate genes, and specifically ACE I/D, on adiposity and BP phenotypes in childhood [9-13]. Such studies may prove insightful for the development of the obesity-hypertension phenotype early in life.

It is well-known that a positive relationship exists between several measures of body size and adiposity (e.g., body mass index, skinfold thickness, etc.) and BP across the lifespan [14]. Similar to other papers, we showed that fatness was significantly related to BP in children, and the differences were most pronounced at the extremes (normal weight vs. overweight) as indicated by a 3.5 times odds of having elevated BP in overweight compared to normal weight youth [15]. Similar to other studies, there was considerable variation in BP among individuals with similar levels of adiposity. It thus seems reasonable to hypothesize that genetic factors modulate the relationship between adiposity and BP [16]. Indeed, recent studies in adults indicate that specific genetic markers modify the relationship between adiposity and BP [7,17,18]. In a previous paper, we found that a positive family history of coronary heart disease (CHD) did not modify the relationship between adiposity and BP in young children [15]. In this study, we replace self-reported family history of CHD with the ACE I/D genotype.

Given the paucity of studies in this emerging area of research, the purpose of this study was to twofold: 1) examine the differences in adiposity and BP between ACE I/D genotypes among young children, and 2) examine if the association between adiposity and BP was modified by the ACE I/D genotype.

## Methods

### Participants

The participants in the study were young children (n = 152, 75 females, 77 males; 86% Caucasian) ages 3–12 years from a rural U.S. Midwestern community (pop. 30,000). The participants were recruited through written and/or verbal advertisements. Parental consent was obtained from all participants, and the procedures were also explained to the child participants. The study protocol was approved by the University of Nebraska at Kearney Institutional Review Board.

### Anthropometry

Stature and body mass were measured according to standard procedures [19]. Stature was measured to the nearest 0.1 cm using a wall stadiometer, and body mass was measured to the nearest 0.1 kg using a standard balance beam scale. The body mass index (BMI) was calculated using the following equation: body mass in kg/stature in m^2^.

### Dual energy x-ray absorbtiometry (DXA)

Whole-body DXA scans were performed with the subject in light clothing while lying supine using a Lunar DPX-L densitometer (Lunar Radiation Corporation, Madison, WI, USA). Percent body fat (%BF), fat mass (FM) and fat-free mass (FFM) were determined using the pediatric medium scan mode (software version 1.5d). The Lunar DPX-L densitometer has previously been cross-validated using the pig carcass in the pediatric weight range [20]. Reliability between scans was achieved using a phantom calibration.

In addition to using the continuous variable of fatness in the statistical analysis, children were classified into one of two groups (normal fat or overfat) based on the cut-points for % body fat used in the FITNESSGRAM test assessment program [21] as determined by a previous study which linked these cut-points for body fatness with significant risk for elevated BP and other CVD risk markers [22]. These cut-points correspond to body fat ≥25% in boys and ≥30% in girls as overfat, and <25% and <30% in boys and girls, respectively, as normal fat.

### Measurement of blood pressure

Resting BP was measured in accordance with standard procedures and recommendations [23]. Appropriate cuff size was determined by measuring the circumference of the right upper arm at its largest point. Resting systolic (SBP) and diastolic (DBP) blood pressures were measured by auscultation after the subject was seated for 10 minutes. The mean arterial pressure (MAP) was calculated as: (SBP-DBP/3) + DBP. Three measurements were taken at 1-minute intervals, and the mean of the three values were used for data analysis. Each technician was trained by the same investigator (KH).

### ACE I/D gene polymorphism

Two single nucleotide polymorphisms (SNP), rs4341 and rs4343, previously found to be in complete linkage disequilibrium with the ACE I/D polymorphism were selected to design polymerase chain reaction-restriction fragment length polymorphisms (PCR-RFLP) [24]. This method was chosen because previous work in our lab has shown that conventional PCR methods (including those by Rigat et al. [5] and Shanmugam et al. [25]) and a new deletion-specific PCR amplification test (developed by Glenn et al. [24]) gave inconsistent results in our sample. Specifically, there were a total of 114 genotypes that were considered consistent for the ACE I/D, and 43 were determined to be problematic using these methods.

Buccal cell samples were collected from participants using Whatman sterile foam tipped applicators and Whatman Indicating FTA^® ^cards and prepared for DNA analysis according to manufacturer's instructions [26]. With the assistance of the Whatman technical support services, the decision was made to switch the final rinse of the punch from TE^-1 ^buffer to water in an attempt to prevent any inhibition of the PCR by ethylenediamine tetraacetic acid. The PCR amplifications were implemented using one 2.0 mm punch from a Whatman Indicating FTA^® ^card, 1 × GoTaq PCR buffer, 0.125 mM of each dNTP, 0.25 mM of each primer and 0.25 U GoTaq DNA polymerase (Promega, Madison, WI, USA) in a 10 μl reaction volume. The PCR conditions were as follows, 94°C for 3 min, 35 cycles of 94°C 30 sec, annealing for 30 sec and 72°C 30 sec, and a final extension at 72°C for 5 min in a PTC 200 thermocycler (MJ Research, Watertown, MA, USA).

Methodology including annealing temperatures, genomic locations and primer sequences of ACE I/D and SNPs, as well as the restriction enzymes and their fragment sizes for PCR-RFLP tests and determination of ACE I/D genotype can be found elsewhere [24]. Digestions were performed according to the manufacturer's instructions (New England Biolabs, Beverly, MA, USA), and the resulting fragments were separated by electrophoresis on agarose gel and visualized using ethidium bromide and UV light. Haploview was used to analyze the ACE I/D and SNP genotypes and to determine Hardy-Weinberg (HW) equilibrium status [27]. The combinations of SNP genotypes used to determine ACE I/D genotype were as follows: DD: rs4341 G/G polymorphism and rs4343 G/G polymorphism; ID: rs4341 C/G polymorphism and rs4343 A/G polymorphism; II: rs4341 C/C polymorphism and rs4343 A/A polymorphism.

### Statistical analysis

Descriptive statistics were calculated for all variables. Sex differences were examined by independent t-test for continuous variables. Analysis of covariance (ANCOVA), controlling for age and sex, was used to examine differences in adiposity; and controlling for age, sex, and height to examine differences in BP variables across genotypes. These covariates were chosen since we included males and females in the analytic sample and the sample varied in age (3–12 years old). Height was included as a covariate in the analysis concerning BP since it is known to influence BP independent of age and sex. Post-hoc tests were performed to detect specific genotype group differences when a significant main effect for genotype was found from the one-way ANCOVA. The associations between adiposity and BP were examined using partial correlation coefficients, controlling for chronological age, sex, and height. The difference in the magnitude of the correlations between genotypes was tested using the Fischer z-transformation.

To test the combined influence of ACE I/D genotype (II or D carrier) and body fatness (normal fat or overfat as previously defined) on BP, four groups were created: ACE II genotype and normal fat, ACE II and overfat, ACE D carriers and normalfat, and ACE D carriers and overfat. To test the combined influence, a one-way ANCOVA was conducted with BP measures (SBP, DBP, MAP) as the dependent variable testing for the main effect of combined genotype/body fatness group while controlling for the covariates age, sex, and height. Post-hoc tests were performed to detect specific genotype/fatness group differences when a significant main effect for combined genotype/body fatness was found from the one-way ANCOVA. A p-value of 0.05 was used for statistical significance. Statistical analyses were conducted using SPSS version 16.0.

## Results

Table 1 provides the descriptive statistics and genotypic frequencies for the sample. Age and body size (height, weight, and BMI) did not differ by sex. Mean height and weight of both boys and girls approximated the 50^th ^percentile of the CDC growth charts. The mean BMI of boys approximated the 50^th ^percentile, whereas the mean value for girls approximated the 75^th ^percentile. There were significant sex differences for % body fat and BP, as girls had higher % body fat and DBP and MAP (p < 0.05). The frequency of ACE I/D genotypes did not differ by sex with the ID genotype being the most frequent (40.1%). The ACE I/D genotypes were not found to be in HW equilibrium (p < 0.022) for this sample of children.

**Table 1:** Physical characteristics of the sample

**Characteristics**	**Males (n = 77)**	**Females (n = 75)**	**Total (n = 152)**
Age (yrs)	7.3 (2.0)	7.4 (2.1)	7.3 (2.0) 2.9–11.9
Ht (cm)	124.0 (13.0)	123.8 (13.3)	123.9 (13.1) 97.2–161.5
Body mass (kg)	25.4 (7.8)	26.3 (8.9)	25.9 (8.3) 13.6–60.1
BMI (kg/m^2^)	16.2 (2.4)	16.7 (2.8)	16.5 (2.6) 10.7–29.1
% body fat	24.0 (7.3)*	29.6 (8.0)	26.8 (8.1) 11.7–51.6
SBP (mmHg)	104.0 (8.4)	105.4 (9.0)	104.7 (8.7) 88.0–133.0
DBP (mmHg)	68.7 (6.8)*	71.2 (6.9)	70.0 (6.9) 56.0–100.0
MAP (mmHg)	80.5 (6.4)*	82.6 (6.8)	81.5 (6.7) 69.3–111.0
ACE D/D, n (%)	23 (29.9)	23 (30.7)	46 (30.3)
ACE I/D, n (%)	29 (37.6)	32 (42.6)	61 (40.1)
ACE I/I, n (%)	25 (32.5)	20 (26.7)	45 (29.6)

Values are mean (SD) for boys and girls, with the total sample also showing the range of values

*P < 0.05 for sex difference

Ht, height; BMI, body mass index; WC, waist circumference; % body fat, percent body fat determined by dual energy x-ray absorbtiometry; SBP, systolic blood pressure; DBP, diastolic blood pressure; MAP, mean arterial pressure

The physical characteristics of the subjects grouped by ACE I/D genotype are shown in Table 2. The physical characteristics of the sample do not differ by genotype, except for body mass and fat-free mass (p < 0.05). On average, individuals with the ACE ID genotype were significantly heavier than those with the ACE II genotype. When dividing the groups by D allele carriers, those individuals possessing a D allele were heavier than II individuals with body mass, BMI, and fat-free mass being statistically significant (p < 0.05). Resting BP values did not differ by genotype.

**Table 2:** Physical characteristics of the sample by ACE I/D genotype

**Characteristics**	**ACE DD (n = 46)**	**ACE ID (n = 61)**	**ACE II (n = 45)**	**D carriers (n = 107)**
^1^Body mass (kg)	25.5 (0.8)	27.1 (0.7)*	24.4 (0.8)*	26.4 (0.5)#
^1^BMI (kg/m^2^)	16.4 (0.4)	16.9 (0.3)	15.8 (0.4)	16.7 (0.2)#
^1^% body fat	27.3 (1.1)	26.8 (1.0)	26.0 (1.1)	27.0 (0.7)
^1^FM-DXA (kg)	7.5 (0.6)	7.8 (0.5)	6.7 (0.6)	7.7 (0.4)
^1^FFM-DXA (kg)	18.1 (0.3)^a^	19.2 (0.3)^a^	17.9 (0.3)^a^	18.7 (0.2)#
^2^SBP (mmHg)	104.6 (1.1)	104.8 (1.0)	104.5 (1.1)	104.7 (0.8)
^2^DBP (mmHg)	70.5 (0.9)	70.0 (0.8)	69.4 (0.9)	70.2 (0.6)
^2^MAP (mmHg)	81.9 (0.8)	82.6 (0.7)	81.1 (0.8)	81.7 (0.5)

Values are estimated mean (SE) for each group. BMI, body mass index; WC, waist circumference; % body fat, percent body fat determined by dual energy x-ray absorbtiometry; SBP, systolic blood pressure; DBP, diastolic blood pressure; MAP, mean arterial pressure

^1 ^controlled for age and sex

^2 ^controlled for age, sex, and height

* P < 0.05 for group difference between ID and II

# P ≤ 0.05 for group difference between D carriers and II

^a ^P < 0.05 for group difference between ID and both DD and II

Partial correlations between measures of adiposity and BP for the total sample and grouped by genotype are shown in Table 3. In the total sample, correlations between % fat and SBP, DBP and MAP were 0.46, 0.30, and 0.42, respectively. Although not significantly different, the ACE ID genotype had the highest correlations (0.41 to 0.65) compared to the other two genotypes (0.19 to 0.38). When grouped by presence of a D allele, the D carriers showed slightly higher correlations compared to II but the differences were not statistically significant as determined by the Fisher z-transformation.

**Table 3:** Partial correlations, controlling for age, sex, and height, between percent body fat and blood pressure by genotype in young children

	**Total** **(n = 152)**	**ACE DD** **(n = 46)**	**ACE ID** **(n = 61)**	**ACE II** **(n = 45)**	**D carriers** **(n = 107)**
	
	**Percent Body Fat**				
**SBP**	0.46*	0.19	0.65*	0.41*	0.48*

**DBP**	0.30*	0.20	0.41*	0.16	0.34*

**MAP**	0.42*	0.24	0.57*	0.31*	0.46*

*P < 0.05 for correlation.

SBP, systolic blood pressure; DBP, diastolic blood pressure; MAP, mean arterial pressure.

**Figure 1:**
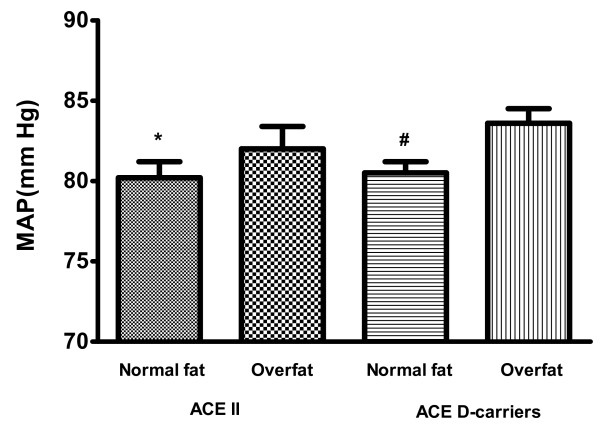
**Differences in mean arterial pressure (MAP) for groups based on combined ACE I/D genotype and body fat category**. * P < 0.05 for difference between II normal fat and D carrier overfat. # P < 0.05 for difference between D carrier normal fat and D carrier overfat.

In general, overfat children had higher BP than normal fat children (SBP: 109.4 ± 1.0 vs 101.6 ± 0.8 mmHg, DBP: 72.7 ± 0.8 vs. 68.2 ± 0.9 mmHg, and MAP: 84.9 ± 1.0 vs. 79.4 ± 0.8 mmHg, respectively; p < 0.05)(data not shown). Approximately 42% of subjects carrying the D allele were overfat compared to 33% in the normal fat group (p = 0.37). When the sample was analyzed by combined ACE I/D genotype and body fat groups, there was a significant main effect on SBP (p = 0.005) (Table 4) and MAP (p = 0.04) (Figure 1). Post-hoc analysis revealed that the high risk group (ACE D carrier and overfat) had higher SBP and MAP compared to the ACE II and normal fat group and the ACE D carrier and normal fat.

**Table 4:** Mean blood pressure values for groups based on combined ACE I/D genotype and body fat category

**ACE genotype**	**II**		**D carriers**	
**Percent body fat category**	**Normal fat** **(n = 30)**	**Overfat** **(n = 15)**	**Normal fat** **(n = 62)**	**Overfat** **(n = 45)**

**SBP (mmHg)**	103.2 (1.3)*	105.7 (1.9)	102.6 (0.9)#	108.1 (1.1)*#

**DBP (mmHg)**	68.6 (1.1)	70.2 (1.6)	69.5 (0.8)	71.4 (1.0)

SBP, systolic blood pressure; DBP, diastolic blood pressure.

* P < 0.05 for difference between II normal fat and D carrier overfat.

# P < 0.05 for difference between D carrier normal fat and D carrier overfat.

## Discussion

Although there is currently considerable interest in the genetics of complex human traits such as BP and other CVD risk factors, few studies have examined the association between candidate genes and CVD risk factors in children. This study provides information on the ACE I/D genotype, adiposity, and BP in a small sample of children. The results indicate that the D-carriers of the ACE I/D genotype show higher BMI and the overfat D-carriers possess higher BP phenotypes compared to the normal fat subjects.

Given the pediatric obesity epidemic and its related co-morbidities including elevated BP, there is considerable interest in the causes and etiology of these phenotypes. Although primarily known for its role in BP regulation, ACE has also been shown to be produced in human adipose tissue [28,29]. A study in mice found that ACE^-/- ^mice weighed less and had less body fat, especially abdominal fat, compared to wild-type mice [30]. Our results in human children show that D carriers were heavier than II individuals with differences in body mass, BMI, and fat-free mass being statistically significant. These results are in accordance with other studies mentioned below that indicate small effects that may be age- and sex-specific. Unfortunately, we could not examine age- and sex-specific differences due to small sample size. In one study, the D allele was associated with higher BMI and waist circumference in 4–6 year old girls that explained 2–4% of the phenotypic variance; but with lower BMI in boys aged 1–2 years that explained 17% of the phenotypic variance [10]. In a study of Greek adolescents (aged 11–18 years), it was found that the D allele was associated with increased subcutaneous fat in females only, and this association was additive with phenotypic values of herterozygotes between those of the two homozygotes [11]. In adults, the association of the ACE genotype and adiposity has been found to be age-related as well. A study of Italian men found the association of BMI and waist circumference with age was significantly larger in individuals with the DD genotype [6]. Furthermore, overweight and abdominal adiposity was found more frequently in men with the DD genotype (OR = 1.8), especially among older men. For men evaluated over 20 years, those with the DD genotype had a larger age-adjusted weight gain (1.5 kg), risk of being overweight (RR = 2.3), and change in diastolic BP (+3 mmHg) compared to the ID or II genotypes [6].

Although the ACE gene is an obvious candidate gene for BP because of its role in the RAA system, results of association studies are equivocal. For example, a meta-analysis of 23 case-control studies found no significant association between the ACE D allele and hypertension (OR 1.10, 95% CI: 0.95–1.27) [31], and a review of studies between 1997 and 2005 involving ACE and hypertension found 12 positive and 14 negative studies in humans [32]. It has been suggested that the equivocal results may be because ACE polymorphisms play a secondary rather than primary role in hypertension [32], or is due to BP being expressed dichotomously (hypertension vs. normotensive) rather than as a continuous quantitative trait [31]. A recent case-control study found that subjects with the ACE DD genotype were 1.6 times more likely to be hypertensive than carriers of the I allele, and that 15% of all cases of hypertension could be attributed to the ACE DD genotype [33]. In both cases and controls, adjusted SBP and DBP were 4.6 and 3.3 mmHg higher in DD individuals than the other two genotypes, respectively [33]. Despite the recent findings, the current evidence indicates any effect of the ACE D allele on the risk of hypertension in adults is small to moderate at best. It is also possible that ACE I/D has more of an influence on ambulatory BP or BP response to stress (exercise or undue stress) rather than resting BP.

Although the results of previous studies in youth are mixed, our results are generally consistent with the majority of these studies showing no significant association between ACE I/D genotype and BP in youth [9,12,34,35]. For example, Bloem et al found no direct significant association between longitudinal BP and ACE I/D genotype [9]. However, they did find an indirect relationship as the ACE I/D genotype was associated with plasma ACE activity which in turn was associated with both mean longitudinal SBP and DBP [9]. Thus, future association studies involving ACE I/D genotype and BP should also measure plasma ACE levels, since the ACE I/D genotype only serves as a proxy for ACE levels and this relationship may not be strong in each individual. On the other hand, studies showing a positive association between ACE I/D genotype and BP in youth reveal the association may be sex-dependent [13,36,37]. A six-year longitudinal study of more than 3500 6–18 year olds found that boys with the ACE DD genotype had significantly higher weight-adjusted SBP than the other ACE genotypes [13]. In a case-control study of hypertension in adolescents, a significant dominant effect of ACE D alleles on SBP was found in boys only [36]. These results mirror those in adults, which show the association between BP and the ACE I/D genotype may be sex dependent [31,38]. Unfortunately, our sample was not large enough to conduct sex-specific analyses.

Another purpose of this paper was to examine if the ACE I/D genotype modified the relationship between adiposity and BP. Our results showing a moderate correlation between adiposity and BP and differences between overfat and normal fat youth confirm previous work [15,39]. However, there remains unexplained phenotypic variation and considerable variation in BP among individuals with similar levels of adiposity. To our knowledge, this is the first study to examine if the association between adiposity and BP is modified by ACE genotype (or any other candidate gene) in children, although a recent study of 292 eight-year-old children found that the magnitude of the association between adiposity and insulin resistance and triglycerides was stronger in ACE DD subjects compared to II or ID subjects [40]. Altered levels of ACE caused by obesity have been previously suggested as a potential pathway through which obesity leads to the elevation of BP in adults [41]. A case-control study in adults found that the DD genotype had 2.5-fold odds of hypertension compared to the II group [42]. However, the additive effects of the ACE D allele and BMI increased the proportion of hypertensive individuals from 40% in non-obese II and ID individuals to 60% in the non-obese DD group and 86% in the obese DD group [42]. Our results offer some confirmation of these findings in that youth who possessed the D-allele and were overfat had significantly higher BP compared to the normal fat youth in either genotype. Thus, it appears that obesity may enhance the expression of ACE I/D genotype differences and lead to elevated BP and perhaps the metabolic syndrome.

## Conclusion

The role of ACE I/D genotype on adiposity and BP phenotypes of children are important to consider in the context of complex, multi-factorial phenotypes. First, these traits are not monogenic, and therefore other candidate genes influence these traits as well. Second, adverse exposure to other environmental factors may also be important to consider. However, we did show that BP was adversely influenced in the overfat D-carriers. Finally, it is possible that ACE I/D may influence these traits differently at various lifestages. Given the paucity of data in the area of genetics and pediatric health and the relative importance of understanding the role of the genome in human health and disease, additional study is warranted in this emerging field of study.

## Competing interests

The authors declare that they have no competing interests.

## Authors' contributions

JCE has made substantial contributions to all aspects of this paper including acquisition of funding; conception and design, acquisition of data, analysis and interpretation of data; and writing the manuscript. MAS has made contributions to the analysis and interpretation of data, and assisted in drafting the manuscript and revising it critically for important intellectual content. KG carried out the molecular genetic studies and drafted the methods section for the genotyping. MFR provided insight into the conception and design of the study, and provided critical feedback to the manuscript for important intellectual content. KAH was responsible for Acquisition of funding, coordinating data collection, and provided critical feedback to the manuscript for important intellectual content. All authors read and approved the final manuscript.
